# Tumor targeted delivery of doxorubicin in malignant peripheral nerve sheath tumors

**DOI:** 10.1371/journal.pone.0181529

**Published:** 2018-01-05

**Authors:** A. B. Madhankumar, Oliver D. Mrowczynski, Becky Slagle-Webb, Vagisha Ravi, Alexandre J. Bourcier, Russell Payne, Kimberly S. Harbaugh, Elias Rizk, James R. Connor

**Affiliations:** Department of Neurosurgery, Pennsylvania State University College of Medicine, Hershey, PA, United States of America; Shanghai Jiao Tong University School of Medicine, CHINA

## Abstract

Peripheral nerve sheath tumors are benign tumors that have the potential to transform into malignant peripheral nerve sheath tumors (MPNSTs). Interleukin-13 receptor alpha 2 (IL13Rα2) is a cancer associated receptor expressed in glioblastoma and other invasive cancers. We analyzed IL13Rα2 expression in several MPNST cell lines including the STS26T cell line, as well as in several peripheral nerve sheath tumors to utilize the IL13Rα2 receptor as a target for therapy. In our studies, we demonstrated the selective expression of IL13Rα2 in several peripheral nerve sheath tumors by immunohistochemistry (IHC) and immunoblots. We established a sciatic nerve MPNST mouse model in NIH III nude mice using a luciferase transfected STS26T MPNST cell line. Similarly, analysis of the mouse sciatic nerves after tumor induction revealed significant expression of IL13Rα2 by IHC when compared to a normal sciatic nerve. IL13 conjugated liposomal doxorubicin was formulated and shown to bind and internalized in the MPNST cell culture model demonstrating cytotoxic effect. Our subsequent *in vivo* investigation in the STS26T MPNST sciatic nerve tumor model indicated that IL13 conjugated liposomal doxorubicin (IL13LIPDXR) was more effective in inhibiting tumor progression compared to unconjugated liposomal doxorubicin (LIPDXR). This further supports that IL13 receptor targeted nanoliposomes is a potential approach for treating MPNSTs.

## Introduction

Neurofibromas are benign nerve sheath tumors in the peripheral nervous system having the potential for transformation into malignant peripheral nerve sheath tumors (MPNSTs). MPNSTs are an aggressive form of nerve sheath tumor also categorized as a soft tissue sarcoma. The estimated lifetime risk of development of MPNSTs in neurofibromatosis type 1 (NF1) patients is 8–13%, compared to 0.001% in the general population[1]. MPNST is a term coined to represent several tumors including malignant schwannoma, malignant neurilemmoma and neurofibrosarcoma, for the tumors of neurogenic origin. There are certain non NF1 MPNSTs which are equally as aggressive as NF1[2]. MPNSTs are an invasive form of soft tissue sarcoma which have a metastasizing property[3–6]. Despite significant advances made in our understanding of the molecular mechanisms related to the development of these benign and malignant tumors, effective systemic treatments have not yet been established. Current treatment methods for peripheral nerve tumors include surgery or non-operative management through the use of radio- or chemotherapy[7]. Therapeutic strategies for the benign lesions utilize anti-angiogenic factors, anti-fibrotic agents, anti-inflammatories and farnesyl transferase inhibitors. While trials are ongoing, none of these strategies to date has shown clear benefit[8]. Doxorubicin is an anthracycline drug and is a chemotherapeutic agent used in certain aggressive MPNSTs[9–11]. However, doxorubicin and its metabolites elicit cardiotoxicity at a high dose and there is a need for the development of a more effective delivery system.

IL13Rα2 is a cancer associated receptor evidenced to be expressed in several malignant tumors including GBM, pancreatic, liver and renal cell cancers[12–15]. Several studies utilize this receptor as a marker for malignant tumors[16–18]. IL13Rα2 is established as an oncogenic receptor and plays a significant role in tumor cell migration, invasion and anti-apoptotic activity[19, 20]. A recent study also demonstrated the inherent potential of IL13Rα2 receptor expressed in cancer cells to protect them from apoptosis, and subsequently decreasing the expression level of this receptor enhances the apoptosis of GBM cells[19]. Drug resistance associated with cancer cells expressing IL13Rα2 may be due in part to the anti-apoptotic nature of this receptor. The expression of this receptor in other normal tissues is negligible with the exception of the testes which expressed the mRNA for IL13 receptor as documented previously[21].

Based on our previous studies using the GBM tumor model, it is evident that IL13 conjugated liposomal doxorubicin is effective in suppressing tumor progression and improving the survival of tumor bearing mice[22, 23]. Thus, in the current study we interrogated the expression of IL13Rα2 in peripheral nerve sheath tumors and in cells cultured from MPNSTs [24, 25]. Our study was then further designed to target MPNST cancer cells using IL13 conjugated liposomes to selectively target and deliver the encapsulated chemotherapeutic agent to improve its therapeutic index and decrease tumor progression in mice bearing MPNSTs.

## Materials and methods

### Ethics statement

Written informed consent was obtained from the patients using a consent form approved for performing this study by Institutional Review Board (IRB) of Penn State Hershey Medical Center and Pennsylvania State University College of Medicine. Signed consent forms were maintained according to the University guidelines following an IRB approved protocol for this study. We have the following IRB numbers for obtaining the tissues from the patients and using them for our studies respectively: IRB protocol # 30750NHR approved on 3-26-09 and IRB #21561EP approved on 8-5-14.

Prior to our studies all our animal experiments were approved by Institutional Animal Care and Use Committee (IACUC) of Pennsylvania State University College of Medicine. The animal health monitoring veterinary care and housing was provided by Penn State Hershey Comparative medicine Department and is in coherence with AALAS and IACUC protocols.

### Materials

1,2-dipalmitoyl-sn-glycero-3-phosphocholine (DPPC), 1,*2*-distearoyl-*sn*-glycero-3-phosphoethanolamine-N-[carboxy(polyethylene glycol)-2000] (ammonium salt) (DSPE-PEG2000), cholesterol, 1,2-distearoyl-*sn*-glycero-3-phosphoethanolamine-N-[maleimide(polyethylene glycol)-2000] (ammonium salt), were purchased from Avanti Polar Lipids. Chloroform and methanol were purchased from Sigma chemicals. Sephadex G25 M was from Amersham Pharmacia, Sepharose CL-2B column was from Sigma Chemicals. IL13Rα2 receptor antibody (Cat No. ab55275) was from Abcam Inc. The mouse strain used in this study is NIHIII Nude mice from Charles River Laboratories. The NF-1 cell line, sNF96.2, was from ATCC (American Type Culture Collection) and the ST88-14 cell line is a gift sample from Dr. Abhijit Guha at University of Toronto, Canada. Cell line #215 was established by culturing the cells from a MPNST excised from an NF-1 patient at Penn State Hershey Medical center and pathologically identified as NF-1 tumor (IRB # 30750NHR approved on 3-26-09 and IRB # 21561EP approved on 8-8-13). STS26T MPNST cells were obtained as a gift from Dr. Daniel Scoles, University of Utah, and later stably transfected with the luciferase vector, which allowed us to monitor the tumor growth in a non-invasive way. All the animal work was approved by the animal care and use committee of the Pennsylvania State University College of Medicine.

All the cell lines were cultured in Dulbecco’s Modified Eagle Medium (DMEM) supplemented with 10% fetal bovine serum. The cell lines were maintained at 37°C in a humidified incubator with 5% CO_2_.

### IL13Rα2 receptor expression in MPNST cells

In order to determine the expression of IL13Rα2 in the MPNST tumors, cell lysates from ST88-14, sNF96.2, STS26T and #215 cells were subjected to polyacrylamide gel electrophoresis (PAGE) using a 4–20% gradient gel. Subsequently, the proteins from the gel were transferred to PVDF membrane using standard Western blotting in a transfer equipment (Biorad, Inc) maintaining conventional voltage, followed by immunoblotting with 2 μg/mL of 1° IL13Rα2 antibody (Abcam, Inc) for 1 hour at room temperature. The membrane was then treated with HRP conjugated anti-mouse secondary antibody. The protein bands in the transferred blots were visualized after treating them with a chemiluminescent substrate. The images were captured using Fujifilm intelligent dark box (Model LAS-3000, FUJI, Inc.).

### IL13Rα2 receptor expression in human tissues

Following IRB approval, human tissue samples from the resection of tumors or the tumor biopsies from patients admitted to the Penn State Hershey Medical Center were frozen at -70°C. A portion of these frozen tissues were sectioned to 10μm using a cryostat and subjected to immunohistochemistry to determine the IL13Rα2 expression. To perform this study, the sections were fixed with 4% paraformaldehyde for 20 minutes and washed with PBS for 5 minutes. The non-specific binding sites on the tissues were blocked with 5% normal goat serum for 30 minutes at 37°C in an incubator. Subsequently, the cells were exposed to anti-IL13Rα2 antibody for 60 minutes at 37°C and washed with phosphate buffered saline (PBS) three times for 5 minutes each wash. The sections were treated with secondary Alexa Fluor 488 conjugated anti-mouse IgG for 60 minutes and then washed with PBS 3 times before gel mounting and viewed under a fluorescence microscope (Nikon Eclipse 80i). A portion of the tissue was thawed in RIPA buffer in the presence of a protease inhibitor cocktail at room temperature and homogenized (IKA Ultraturrax T25 homogenizer) at 4° and centrifuges at 13000g for 10 minutes to collect the homogenates for immunoblots.

### IL13Rα2 expression in tissue microarray

Peripheral nerve carcinoma tissue microarray was obtained from US biomax, Inc., MD (Catalog No. S01001) containing 22 cases of malignant tumors (20 malignant schwannoma, originating from various organs such as fibrous tissue, mediastinum, stomach, retroperitoneum, nerve, soft tissue,pelvic cavity and 2 primitive neuroectodermal tumors), 20 cases of benign tumors (12 neurilemmoma, 2 ganglioneuroma and 6 neurofibroma), 1 cancer adjacent to normal tissue and 7 normal nerve tissue. Duplicate cores per case were available in the microarray. The immunohistochemistry on the tissue microarray was performed using VECTASTAIN ABC systems (Vectorlabs, Inc, CA, USA) following manufacturer’s instruction. Briefly, the tissue sections were deparaffinized and antigen retrieval was performed. After blocking the non-specific binding sites using normal goat serum, the tissue microarray was treated with primary antibody for human IL13Rα2 (mouse IgG1) (Diaclone, Inc., France, Cat. No. 852.12.000) followed by treatment with biotin conjugated secondary antibody, followed by color development using DAB (3, 3’-diaminobenzidine) kit.

### Tumorigenic potential of IL13Rα2 expressing MPNST cells

To determine the tumorigenic potential of the IL13Rα2 expressing ST88-14 cells, we injected 1x10^5^ ST88-14 cells in the sciatic nerve of NIHIII nude mice (Crl: NIH-*Lyst*^*bg-J*^*FOXn1*^*nu*^*Btk*^*xid*^). In addition to lacking a thymus and T-cell function, NIH III nude mice also have x-linked immunodeficiency (xid) and beige (bg), which results in T-independent B-lymphocyte and natural killer (NK) cell deficiency, respectively. For the sciatic nerve injection procedure, the mouse was anesthetized with ketamine/xylazine mixture and a 1.5 cm skin incision was made over the proximal hind limb just posterior to and parallel with the femur. Blunt dissection was then carried out through the superficial muscles of the hind limb until the sciatic nerve was visualized. The tumor cells were then injected using a Hamilton syringe (22 gauge needle). The tumor was allowed to grow for 8 weeks, after which the animal was sacrificed by overdosing with ketamine/xylazine. The sciatic nerves were dissected, removed and sectioned. Normal sciatic nerve in the same animal was surgically removed and sectioned to 10 μm thickness on slides. All tissues were then paraffin embedded. The sections were subjected to IHC for identification of IL13Rα2 following a similar protocol as described above for the human tumor tissue.

### Evaluation of Ki-67 and S100 in the tumor tissues from the sciatic nerve tumor model

Ki-67 cell proliferation marker expression was analyzed in several nerve tumor tissue lysates by immunohistochemistry. In addition, IHC was also performed on the sciatic nerve tumor tissue from the mouse model to confirm the tumor pathology. S100, Ki-67 and IL13Rα2 were all analyzed by IHC. Nerve tumor tissues analyzed included schwannomas, neurofibromas and MPNSTs. The tissues were processed and paraffin embedded sections of 10 μm were obtained. The tissues were deparaffinized and subjected to immunohistochemistry for Ki-67 by exposing the tissues to goat polyclonal antibody for Ki-67 (Cat. No. sc-7844, Santa Cruz Biotechnology, Inc) at a dilution of 1: 200, followed by treatment with Alexa Fluor anti-goat antibody. The nucleus was stained with DAPI, the slides were mounted on coverslips and images were observed and recorded in a CCD camera attached fluorescent microscope.

### Formulation of IL13 conjugated liposomal doxorubicin

IL13 conjugated liposomes encapsulated with doxorubicin were prepared following the methodology reported in our earlier publication[22]. Briefly, the lipids 1,2-dipalmitoyl-*sn*-glycero-3-phosphocholine (DPPC), cholesterol, 1,2-distearoyl-*sn*-glycero-3-phosphoethanolamine-N-[amino(polyethylene glycol)-2000] (DSPE-PEG-2000) and 1,2-distearoyl-*sn*-glycero-3-phosphoethanolamine-N-[maleimide(polyethylene glycol)-2000] (DSPE-PEG2000-Maleimide) were solubilized in chloroform and methanol in the ratio of 1:1 (v/v) in a round bottom flask and a thin film was made in a round bottomed flask using a rotary evaporator (Buchi, Inc). The thin film was dried in nitrogen to remove traces of solvents and reconstituted in ammonium sulfate solution (155mM, pH 5.5). The doxorubicin was encapsulated following the ammonium sulfate gradient method as described previously [22, 23]. The un-encapsulated doxorubicin was removed by passing the liposomes through a Sephadex G25 column (GE Healthcare, Inc, USA). The conjugation of maleimide bearing liposomes with thiolated IL13 was performed as described earlier [22].

### Specific binding and internalization of IL13 conjugated liposomal doxorubicin (IL13LIPDXR)

The binding of the IL13LIPDXR on the MPNST cells expressing IL13Rα2 was analyzed by performing *in vitro* binding and internalization studies in NF-1 cells. ST88-14 and sNF96.2 NF-1 cells were cultured in 8 well chamber slides at a density of 20,000 cells per well and then treated with IL13 conjugated liposomal doxorubicin (IL13LIPDXR) at a concentration of 20μM for 2 hours at 37°C. The cells were then fixed with 4% paraformaldehyde and immunocytochemistry was performed using IL13Rα2 antibody (Abcam, Inc) at 2μg/mL concentration. This was then followed by secondary anti-mouse Alexa Fluor 488 along with DAPI (4',6-Diamidino-2-Phenylindole, Dihydrochloride). These slides were washed with phosphate buffered saline, gel-mounted with glass coverslips and visualized under a confocal microscope (Leica TCS SP2, Leica Microsystems).

### Intraspheroidal transport of IL13LIPDXR in MPNST tumor spheroids

ST88-14 cells were cultured in an anchorage independent manner to form multicellular tumor spheroids following our earlier protocol[22]. Briefly, 24 well plates were coated with 1% seaplaque agarose and MPNST cells were cultured by adding 1x10^5^ cells per well for 3–4 days until a single spheroid in the size range 3–5 μm started forming. The spheroids were transferred to glass bottom dishes (Matek Corporation, Inc, MA, USA) and treated with 20 μM of IL13 conjugated liposomal doxorubicin for 120 minutes at room temperature. The liposome internalization process was visually followed through a confocal microscope. Z-stack images were obtained at various time points for every 15–30 minutes and the maximum projection from the z-stack images at each time interval was recorded.

### Cell proliferation assay on NF-1 MPNST cells cultured as a monolayer

To assess the cytotoxicity of the targeted liposomes in the cell culture model, sNF96.2 cells and ST88-14 cells were cultured in 96 well plates at a concentration of 4x10^3^ cells per well for 24 hours. After a 24 hour incubation period, the cells were treated with IL13LIPDXR at a concentration of 0, 10, 100, 250, 500, 1000 and 2000 ng/mL and incubated for 48 hours. The cells were then treated with Alamar blue reagent (Life Technologies, Inc.) following manufacturer’s instruction. After 6 hours of incubation, the plates were analyzed using a fluorescence plate reader at excitation/emission wavelengths of 560/590nm. The measurement was made in triplicate and the percentage control was measured with respect to the untreated cells.

### In vivo tumor inhibitory effect of IL13LIPDXR

To evaluate the *in vivo* therapeutic efficacy of IL13LIPDXR, sciatic nerve tumor models were generated as detailed previously with STS26T-Luc MPNST cells. Briefly, 50,000 STS26T-luc cells were injected into the sciatic nerve after surgical isolation and the cells were allowed to grow in the nerve for 2 weeks. Intratumoral luciferase activity was determined by a Xenogen instrument (IVIS 200) after an intraperitoneal injection of 2.8 mg/mouse of D-luciferin potassium salt solution. After a 7 minute incubation period, the animals were scanned in the IVIS imager (Perkin Elmer) for 1 minute. A linear relationship between the bioluminescent intensity and the tumor weight is evident by earlier reports, indicating that number of tumor cells contribute primarily to the tumor weight rather than tumor volume[26, 27]. IVIS imaging of tumors is also able to accurately monitor tumor progression over time[28–30]. The mice which demonstrated successful tumor growth after 2 weeks were utilized for the experiments. We normalized the mice into three groups (n = 5 each) based on initial tumor size. These groups included: (a) Mice treated intraperitoneally with targeted IL13LIPDXR at a dose of 7 mg/kg body weight, (b) mice treated intraperitoneally with non-targeted LIPDXR at a dose of 7mg/kg body weight and (c) control mice injected intraperitoneally with saline. The tumor growth was monitored once weekly using the IVIS instrument. The total counts were measured with the IVIS imager. The veterinary care staff inspects the animals daily for any signs of adverse health effects. Mortality was seen in mice towards the end of the study due to tumor growth becoming extensive and inhibiting animal movement, drinking, and feeding. When the sciatic tumor becomes larger to an extent where the animal will be unable to walk, eat or drink and if the animal has lost about 10% of its weight the mice were euthanized for humane reason. To minimize the suffering and distress during surgical procedure, the animals were anesthetized by using isofluorane (inhaler anesthesia) before the surgical procedure. Similarly before performing the IVIS imaging the animals were anesthetized using isoflurane (inhaler anesthesia). The isoflurane was administered at a dose of 2.5–5% induction and 0.5–5% maintenance. When the animal has reached its endpoint, before euthanizing them they were injected with ketamine/xylazine injection at a dose of 100mg/kg/10mg/kg. Before during and after surgery the mice were administered with Buprenorphine at a dose of 0.05–0.1mg/kg body weight by subcutaneous injection. The analysis of tumor volume data was performed with one-way ANOVA using GraphPad prism software.

## Results

### Immunohistochemistry on PNST tissues from patient biopsy and cells indicated the expression of IL13Rα2

**Fig 1:**
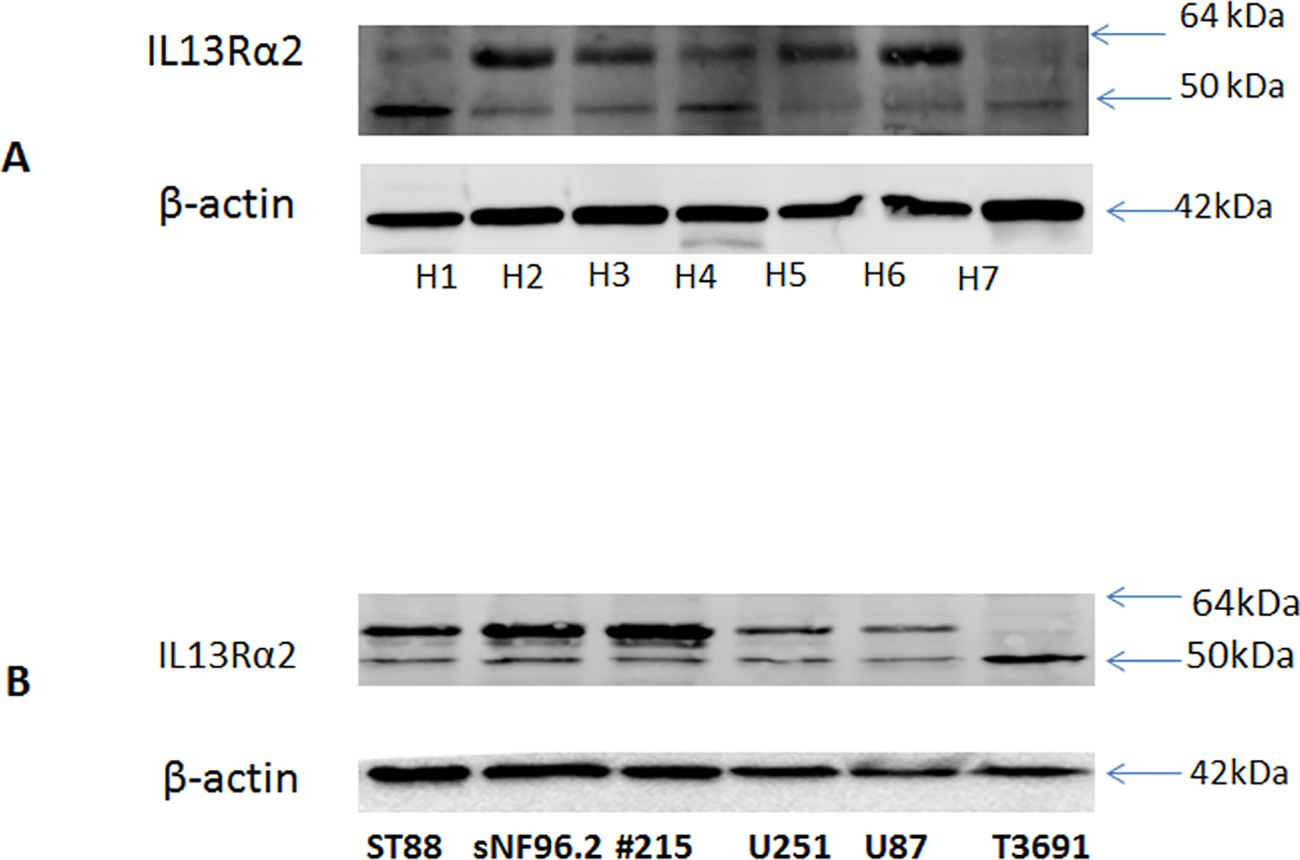
A. Immunoblots showing the expression levels of IL13Rα2 in human tissue homogenates from various nerve tumor tissues: H1- schwannoma, H2-neurofibroma, H3-schwannoma, H4-MPNST, H5-schwannoma, H6-neurofibroma, H7-neurofibroma. B. Human neurofibroma cell lines sNF96.2, ST88-14 and #215 cell lines were all found to express IL13Rα2 as evidenced from the Western blots.

**Fig 2:**
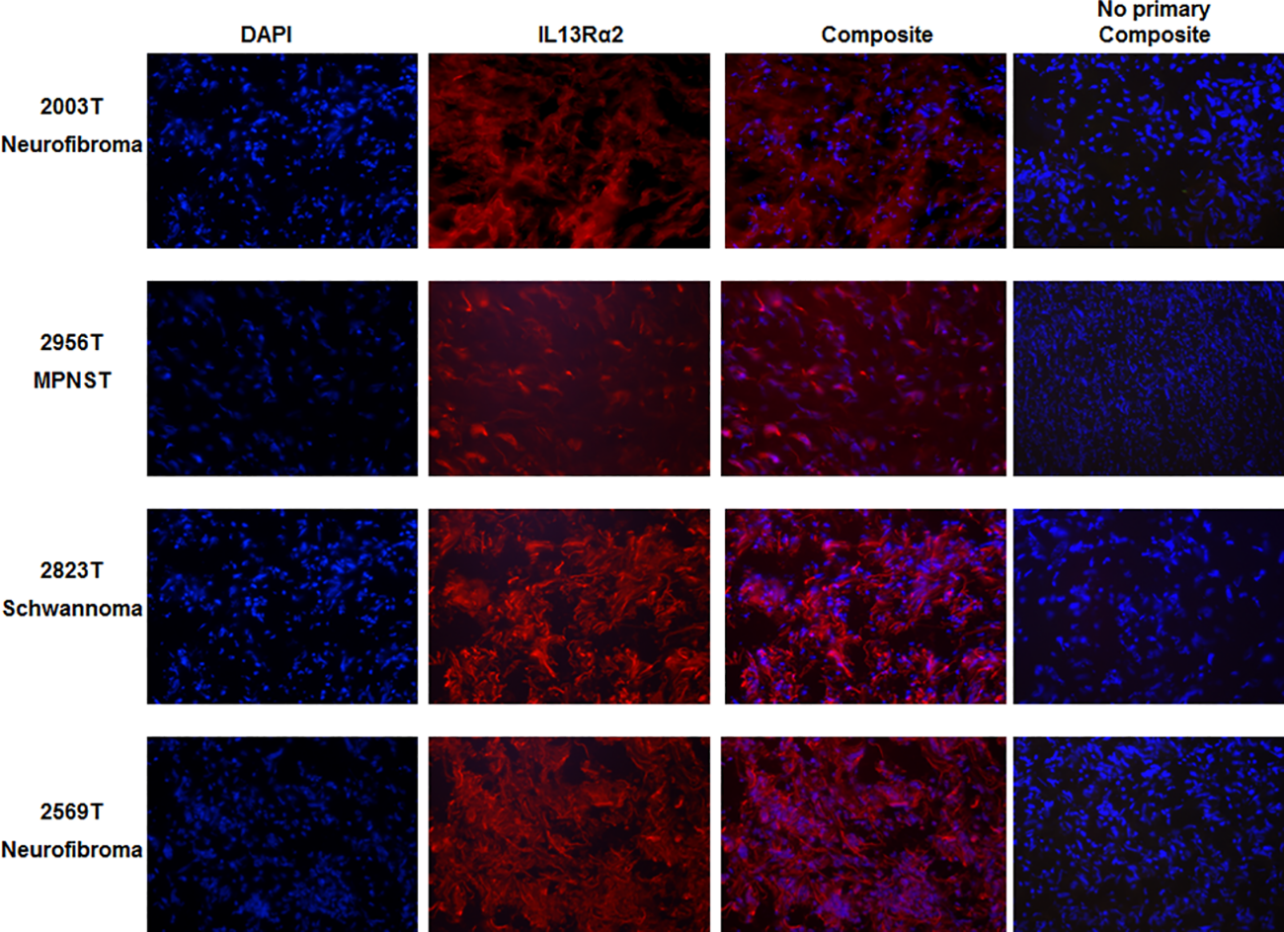
Immunofluorescence of benign and malignant peripheral nerve tumors for IL13Rα2 (red). The blue color represents the nuclear staining with DAPI. IL13Rα2 expression is evident in MPNST, low grade schwannomas and neurofibromas. From the composite images, most of the staining appears to be cytoplasmic as well as nuclear.

**Fig 3:**
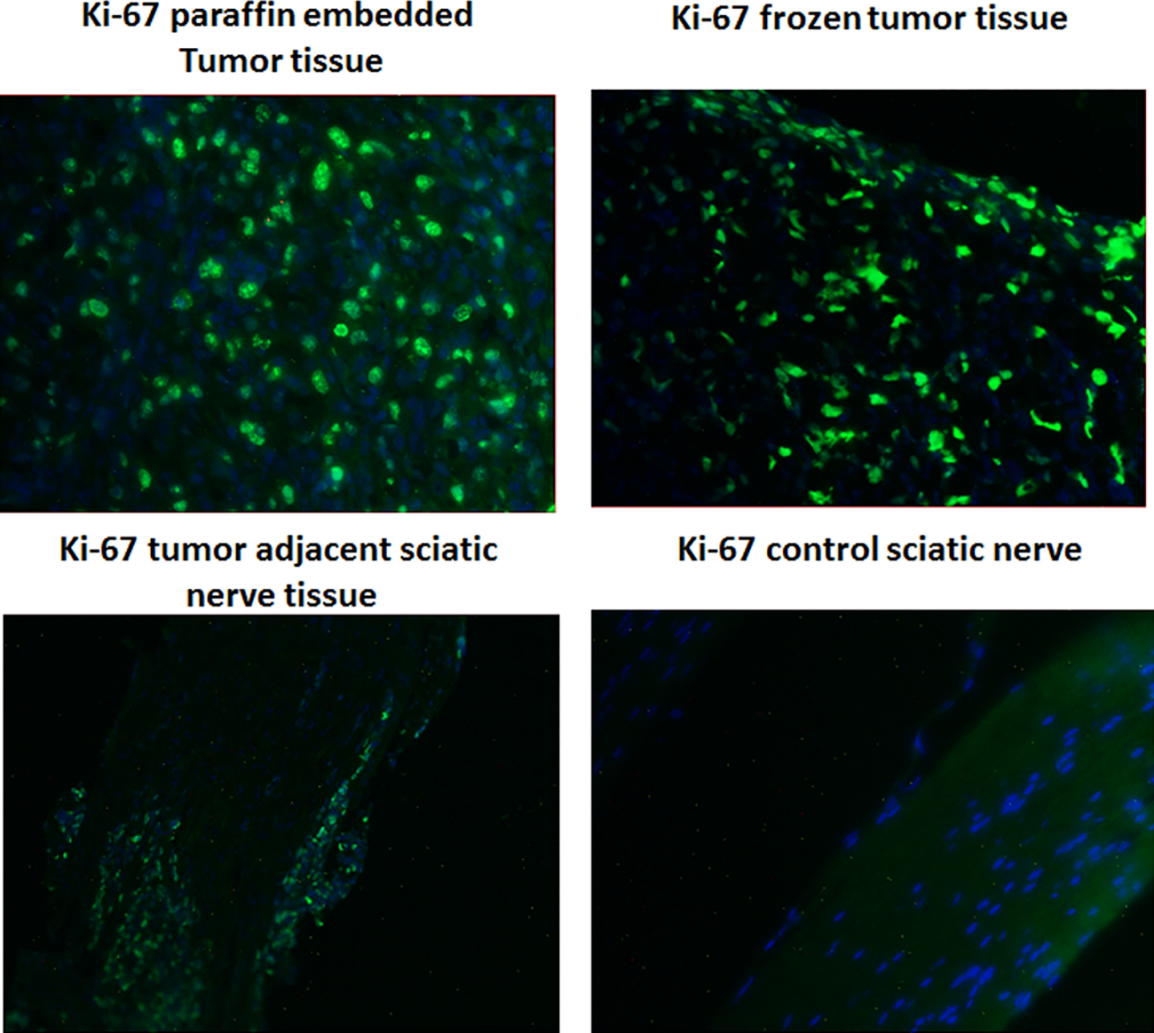
Immunohistochemistry for Ki-67 expression on the sciatic nerves from the tumor induced and control (untreated) mice indicates that the IL13Rα2 staining pattern was intense in tumor tissue.

**Fig 4:**
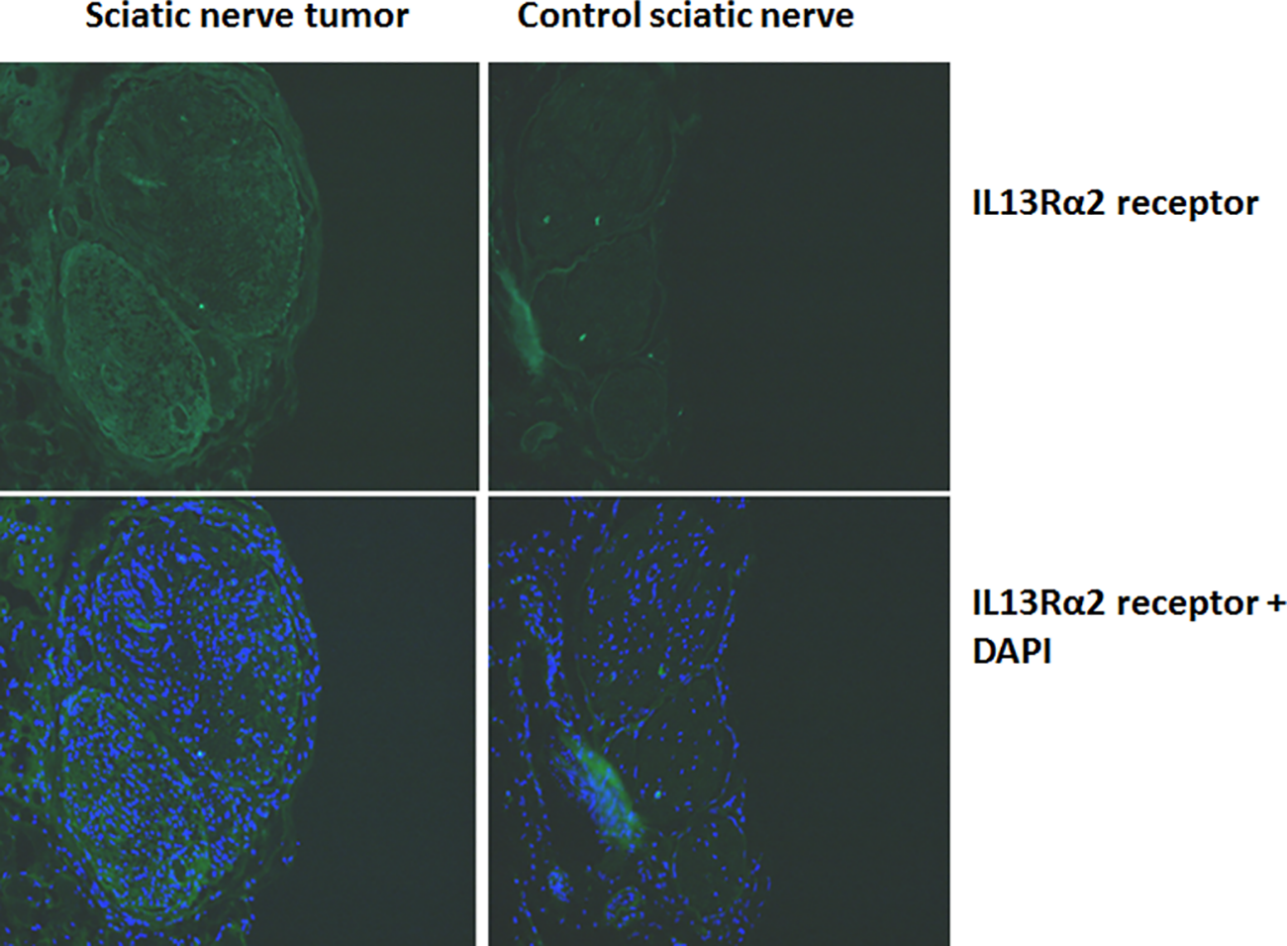
IL13Rα2 expression in the sciatic nerve tumor developed in NIHIII mice injected with sNF96.2 NF-1 cells. The expression levels were compared with the control sciatic nerves which were not injected with the cells.

All 7 peripheral nerve sheath tumors examined for this study robustly express IL13Rα2 receptor (Fig 1A). Investigation by immunohistochemistry (IHC) on an additional 4 peripheral nerve tumors from the Pennsylvania State University core facility indicated that all of them were also positive for IL13Rα2 (Fig 2). The expression levels are not significantly different among the tissues based on IHC (Fig 2). Western blots of cell lysates indicate a double band for sNF96.2, ST88-14 and #215, one at 64 kDa corresponding to glycosylated IL13Rα2 and another at 50 kDa; a non-glycosylated form of the receptor (Fig 1B). The tumor from neurofibromas and schwannomas also express the bands corresponding to glycosylated and non-glycosylated forms of IL13Rα2. Previous work by another group has verified the glycosylation of IL13Rα2 to be a significant event in tumor progression[31]. We have also compared the Ki-67 expression in the malignant and benign peripheral nerve tissues by immunohistochemistry. They all showed Ki-67 expression at variable levels as evident from the supplementary figure S1 Fig (data can be found in the panel A-F in S1 Fig). Immunohistochemistry on sciatic nerve tumor tissue obtained from a tumor induced mouse indicated Ki-67 immunostaining in both normal as well as tumor induced sciatic nerves (Fig 3). IL13Rα2 expression is found predominantly only in the tumor induced sciatic nerve, whereas very weak expression is found in the normal sciatic nerves as evident from immunoblots with the tissue lysates and IHC with the paraffin embedded tissue sections (Fig 4).

### IHC on peripheral nerve carcinoma tissue microarray

**Fig 5:**
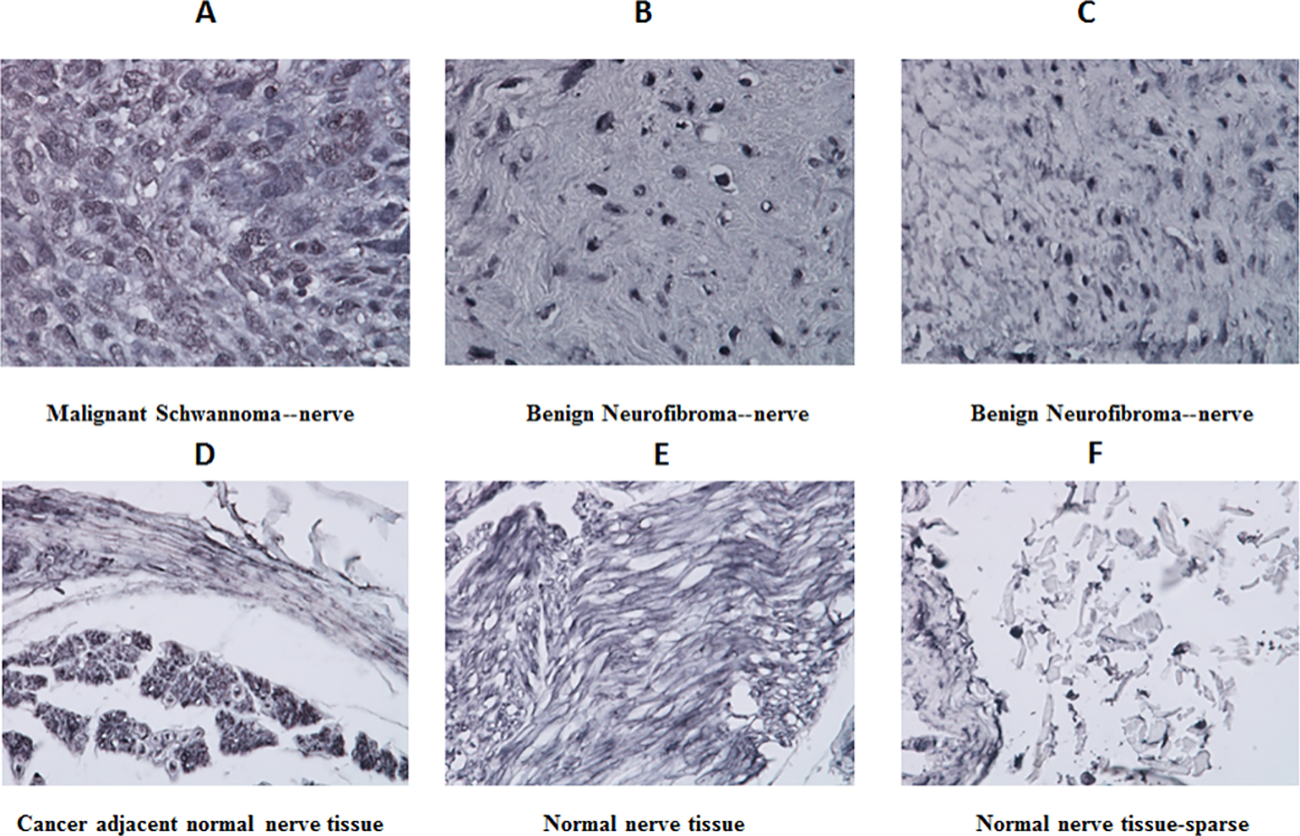
IL13Rα2 expression in various tissues from a peripheral nerve carcinoma tissue microarray. Representative microscopic images from the tissue microarray after IHC for IL13Rα2 expression are shown (panel A-F). Malignant Schwannoma distinctly shows robust expression of IL13Rα2 in the tissue (D) with positive expression in benign neurofibromas as evident from the IHC (E,F). Normal nerve tissue indicated minimal staining without distinct cellular morphology. No IL13Rα2 staining was observed in cancer adjacent normal tissues.

Fig 5 shows representative images from immunohistochemistry performed on the peripheral nerve carcinoma tissue microarray. Among the 22 malignant nerve tissues (in duplicates) that were present in the microarray, 17 were positive for the expression of IL13Rα2 and a representative microscopic image is shown in Fig 5, panel A, which clearly showed the densely populated cells to be stained for the receptor. Also among the malignant tissues, 3 were negative for IL13Rα2 expression and 2 were inconclusive based on duplicate samples. Among the benign nerve tumors, out of the 12 neurilemoma (benign) analyzed, 9 were positive, 1 was negative for IL13Rα2 expression and one was inconclusive. Interestingly, all of the 2 ganglioneuroma (benign) and 6 neurofibroma (benign) that were present in the microarray were positive for IL13Rα2 expression. Representative images from benign neurofibromas are shown in Fig 5, panel B, C. Among the 7 normal nerve controls that were present in the microarray, all of them were either negative or weakly positive for IL13Rα2 expression. An IHC section from such a normal control is shown in Fig 5 (panel E, F). Interestingly, cancer adjacent to normal nerve tissues indicated a distinct demarcation of the boundary between the tumor and normal region. There is an intense nuclear staining in the tumor region, whereas no such staining in the normal nerve region was observed (panel D).

### Specific binding and internalization of IL13 conjugated liposomal doxorubicin to MPNST cells in culture

**Fig 6:**
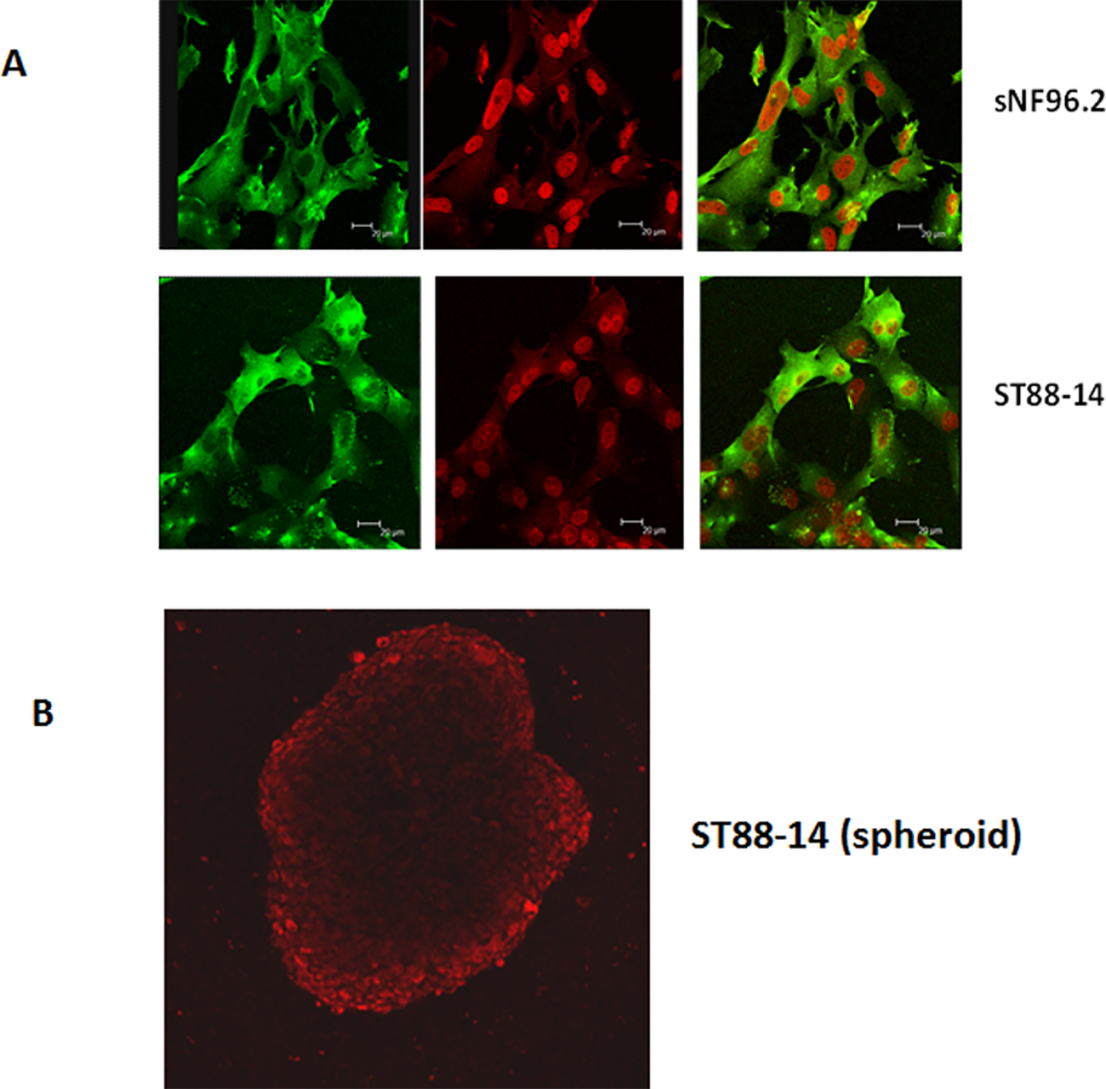
Fluorescent microscopic images of sNF96.2 and ST88-14 MPNST cells after exposure to IL13LIPDXR for 2 hours, followed by immunocytochemistry for IL13Rα2. The green color in the cytoplasmic region represents the expression of IL13Rα2 receptor and the red color represents the endogenous fluorescence of the internalized doxorubicin delivered through targeted liposomes. (B) IL13LIPDXR is also able to bind and internalize through multicellular spheroids after exposure for 2 hours as evidenced by confocal microscopy images. More details can be found in the S2 Fig and S1 Video.

The IL13LIPDXR was able to bind to and be internalized by the MPNST cells (Fig 6A). The intracellular red color indicates the endogenous fluorescence of doxorubicin, while the green fluorescence represents the expression of IL13Rα2 (by immunocytochemistry) in the cell membrane and cytoplasmic region. Based on the endogenous fluorescent property of doxorubicin, the binding and transport of the IL13LIPDXR in the ST88-14 tumor spheroids was monitored by confocal microscopy and the fluorescent image indicates that IL13LIPDXR infiltrated the tumor spheroids. It is also evident that with the increasing time interval, IL13LIPDXR binds and tending to diffuse towards the core of the ST88-14 spheroids (Fig 6B and S2 Fig). The maximum projection from the z-stack images of the spheroids at various time points are shown in supplementary figure S2 Fig (only time points 40, 60, 100 and 120 minutes are shown). As it is difficult to show all the z-stack images from each time point, a video clip was created from the collated z-stack images at various time points up to 120 minutes. This video clip is available as a supplementary file (S1 Video).

### Cytotoxicity assay on the NF-1 MPNST cells cultured in monolayer

**Fig 7:**
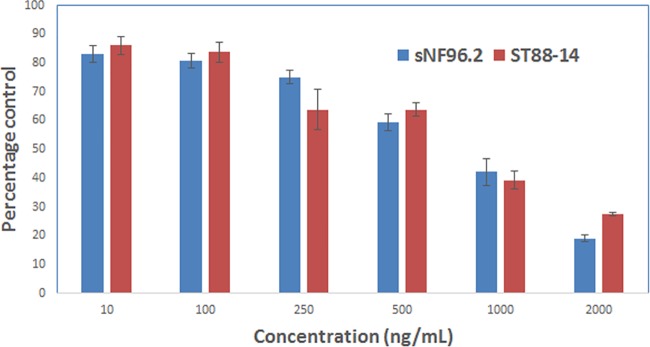
Cell proliferation assay performed on sNF96.2 and ST88-14 MPNST cells cultured in monolayer after treatment with IL13LIPDXR. The results are displayed as percentage control with respect to untreated cells. 48 hours post treatment the cells were stained with Alamar blue (resazurin), a redox dye which exploits the reducing potential of the viable cells, whereby the non-fluorescent resazurin will be converted to a highly fluorescent resorufin, which can be monitored by a fluorescent plate reader.

A dose dependent cytotoxic effect was observed when the cultured ST88-14, STS26T and sNF96.2 cells were exposed to IL13 conjugated liposomal doxorubicin for 48 hours. The cytotoxic effect of IL13LIPDXR observed in both the cell lines, ST88-14 and sNF96.2, was compared to the untreated controls. With increasing concentration of the liposomes, the percentage of viable cells decreased. At a concentration of 2μg/mL, about 70–80% of the cells were killed by the liposomal doxorubicin formulation in sNF96.2 and ST88-14 cells respectively, compared to the untreated controls (Fig 7).

### Targeting IL13Rα2 using IL13LIPDXR attenuates tumor progression in MPNST mouse models

**Fig 8:**
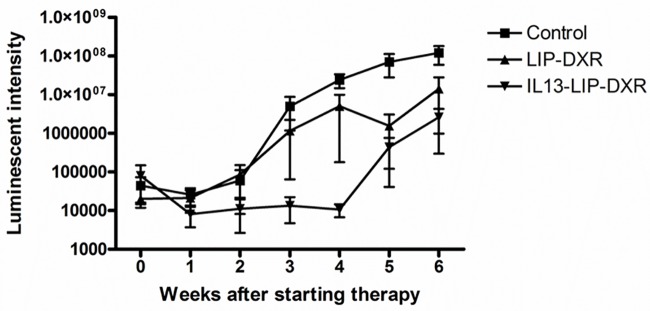
*In vivo* tumor progression in the murine MPNST tumor model after administration of a once per week dose of 7 mg/kg body weight of (a) IL13LIPDXR (b) unconjugated LIPDXR and (c) control mice which were injected with saline.

The effect of targeting IL13Rα2 was investigated in mice with luciferase transfected STS26T MPNST cell induced sciatic nerve xenografts. Delivery of doxorubicin through IP injection of IL13LIPDXR resulted in slower tumor progression when compared to control (saline injected) and unconjugated liposomal doxorubicin (LIPDXR) injected groups (Fig 8). In the IL13LIPDXR group, the tumor progression was slower for 4 weeks beyond which the tumors started growing rapidly. However in contrast, the tumor growth in the control group was extremely rapid even in the initial phase of 4 weeks. Moreover, based on the luminosity values from the Xenogen IVIS instrument, the tumor progression in the control group was increased by several folds when compared to the IL13LIPDXR group (Fig 8) (luminosity was >10^7^). Raw data corresponding to tumor volume is available as supporting document (S1 Raw Data).

## Discussion

IL13Rα2 is a cancer associated receptor known to be expressed in several cancers such as glioblastoma, head and neck, and renal cell carcinoma[32–35]. Earlier studies have demonstrated that IL13Rα2 is a viable therapeutic target for malignant and invasive cancer therapy. Its expression in soft tissue sarcomas like MPNST has not been studied thoroughly. Although the receptor is a non-signaling receptor, its presence and the expression level signifies the malignancy of the cancer and also predicts the invasiveness, metastasis and poor prognosis of certain cancers[36, 37].

In our study we identified the expression of IL13 receptor in malignant and benign peripheral nerve sheath tumors. There is an abundant expression of IL13Rα2 in MPNST tissues derived from human patients and in cell lines. Thus, our study suggests that it may be possible to use IL13Rα2 as a marker protein to identify peripheral nerve sheath tumors and also to distinguish between benign and malignant peripheral nerve sheath tumors. Current pathologic determination of MPNSTs includes immunohistochemistry with S100, Ki-67 and p53[38, 39]. Ki-67 is a proliferating cell nuclear antigen expressed by several proliferating cancer cells and is generally used to distinguish rapidly proliferating cancerous cells from normal cells. In particular, Ki-67 is used widely to distinguish malignant from benign peripheral nerve sheath tumors[40]. Based on our study, IL13Rα2 has a similar property as that of Ki-67 in identifying malignant peripheral nerve sheath tumors and differentiating them from their benign counterparts. Based on the IHC from the sciatic nerve tissue derived from the mouse model, the MPNST tumor showed distinct immunoreactivity for IL13Rα2 compared to the control sciatic nerve.

The identification of this receptor expression can be exploited for therapeutic purposes to deliver drugs and genes through receptor targeted nanovesicles (liposomes) or IL13 based fusion toxins. Therapeutic strategies with bacterial toxins like IL13PE38QQR (Pseudomonas Exotoxin), IL13DT (Diphtheria toxin) and IL13 linked nanovesicles were demonstrated to selectively target and eliminate GBM tumors leading to increased survival of the tumor bearing mice. In our earlier studies, we showed improved efficacy of the IL13 conjugated liposomal doxorubicin (IL13LIPDXR) in GBM tumors expressing IL13Rα2. Doxorubicin is one of the anthracycline drugs used widely for treatment of several malignant solid and soft tissue cancers. Few case studies were reported where doxorubicin alone or in combination with other chemotherapeutic agents were used to treat MPNSTs with moderate efficacy[41–43]. Doxil, a pegylated liposomal doxorubicin, was demonstrated previously to be effective in metastatic soft tissue sarcoma patients [44]. IL13LIPDXR is the formulation similar to Doxil, but with IL13 cytokine attached on the surface PEG groups for improved tumor-specific targeting.

From our present investigation, it is evident that IL13 conjugated liposomes were readily taken up by the IL13Rα2 expressing NF-1 type MPNST cells in monolayer. It is also clear that the cytoplasmic and membrane fraction of the cells express IL13Rα2, which is the driving force for the enhanced binding and internalization process of the liposomes. It is also interesting to observe a nuclear localization of doxorubicin when delivered through the liposomes. Based on our earlier investigation, we attribute this property to the ability of IL13LIPDXR to overcome the multidrug resistance imparted by the cancer cells [23]. The binding and intercellular diffusion of the IL13LIPDXR is also evident from the 3D spheroid culture model, which indicates that the liposome formulation can diffuse into *in vivo* tumors. The cell proliferation assay with ST88-14 and sNF96.2 cells demonstrates the *in vitro* cytotoxic potential of the IL13LIPDXR on MPNST cells in a concentration dependent manner (Fig 7) indicating that doxorubicin can be effectively delivered to these malignant cancer cells. A similar trend is reflected in the *in vivo* experiments. The group of mice bearing MPNST tumors that were treated with targeted liposomal doxorubicin treatment group had a decrease in tumor progression when compared to the non-targeted liposomal doxorubicin treatment group.

**Fig 9:**
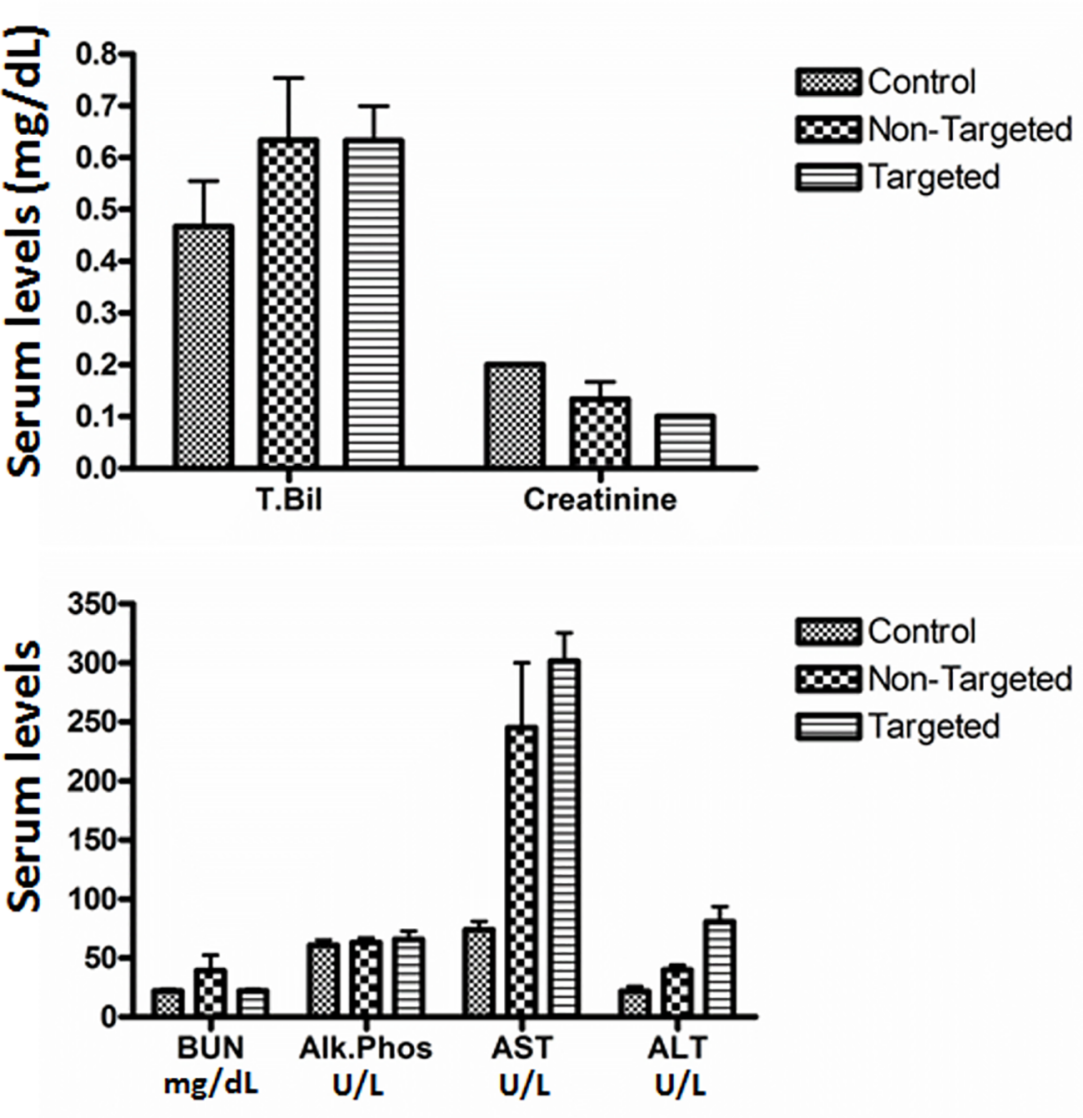
Serum chemistry analysis. The analysis was performed 48 hours post injection of the 7mg/kg dose of targeted and non-targeted liposomal doxorubicin. The values were compared with control mice injected with phosphate buffered saline. The levels indicate that liver functional enzymes including bilirubin and alkaline phosphatase are comparable in both the treated and control groups of mice. Creatinine and BUN levels are also comparable to that of untreated control mice, indicating that renal function is not significantly affected due to treatment.

An in-depth investigation of the mechanism of interaction of the IL13 conjugated nanoliposomes with glioma tumors expressing IL13Rα2 and their ability to overcome the known drug resistance properties was described in our earlier study [23]. Based on our findings of overexpression of this receptor in the malignant peripheral nerve sheath tumors, it appears possible to utilize these liposomes for therapeutic purposes in drug resistant metastatic MPNSTs. Based on our *in vivo* therapeutic efficacy study in the sciatic nerve tumor model, it is evident that tumor progression is attenuated in the mice treated with IL13LIPDXR at 7 mg/kg body weight, when compared to the non-targeted group. Interestingly, it is also obvious from the data that compared to control saline treated mice, the non-targeted liposomal doxorubicin treated mice have slower tumor progression, which demonstrates the partial efficacy due to the liposomal doxorubicin alone. The therapeutic effect seen in the tumor bearing mice is amplified upon targeting the liposomes to IL13Rα2. Enzyme levels of alkaline phosphatase, creatinine, blood urea nitrogen (BUN) and alanine amino transferase (ALT) were analyzed and were comparable to that of control mice (Fig 9) indicating that the treatment does not significantly affect liver and kidney function.

In addition to MPNSTs, certain benign nerve tumors like neurofibromas and schwannomas can also be targeted with this ligand targeted liposomes. In the case of benign nerve tumors, doxorubicin can be replaced with other therapeutic agents including imatinib mesylate (Gleevec) and Ras inhibitors shown previously to be effective in treating and inhibiting malignant transformation of such tumors[45, 46]. The IL13Rα2 expression level is lower in these benign tumors than that of the MPNSTs, but it is still present in detectable amounts, suggesting the opportunity to target those benign tumors with IL13 conjugated liposomes for efficacious therapy.

## Conclusions

Expression of IL13Rα2 in malignant and benign peripheral nerve sheath tumors is evident in several tissues and cell lines, indicating this receptor may function as a potential target for receptor targeted therapy. Our *in vitro* and *in vivo* experiments with peripheral nerve sheath tumor cells and animal models clearly suggest that targeting the IL13Rα2 with IL13 conjugated liposomes will increase the accumulation of the drug in the tumor and subsequently decrease the tumor burden.

## Supporting information

S1 Fig Ki-67 proliferation index marker expression in various peripheral nerve tissue lysates and control samples. Ki-67 expression is evident in the neurofibromas, schwannomas and MPNSTs at variable levels (panel A-F). The Alexa Fluor 488 (green) staining represents the expression of Ki-67 and the DAPI (blue) stains the nucleus.
(TIF)

S2 Fig *In vitro* binding and intracellular diffusion of IL13LIPDXR in the ST88-14 multicellular tumor spheroid. The figure indicates the projection maximum from the z-stack images at various time intervals. The endogenous fluorescence (red) of the IL13LIPDXR was utilized here to probe the intraspheroidal transport and diffusion into the cells.
(TIF)

S1 Raw Data The raw data from the serum chemistry analysis of the mice after treatment (Table 1A &1B) and the signal intensity from the IVIS instrument, which corresponds to tumor volume (Table 1C). (DOCX)

S1 Video The z-stack images from confocal microscopy of the ST88-14 tumor spheroids after exposure to IL13LIPDXR at various time points were compiled to a video file which is available as a supplementary file: https://www.dropbox.com/s/g92vde6ni9mf0rc/LIPST88.wmv. (WMV)
