# Supplementary figures and images for: Tumor targeted delivery of doxorubicin in malignant peripheral nerve sheath tumors
Source: PLoS One. 2018 Jan 5;13(1):e0181529. doi: 10.1371/journal.pone.0181529 (PMC5755733; doi:10.1371/journal.pone.0181529)

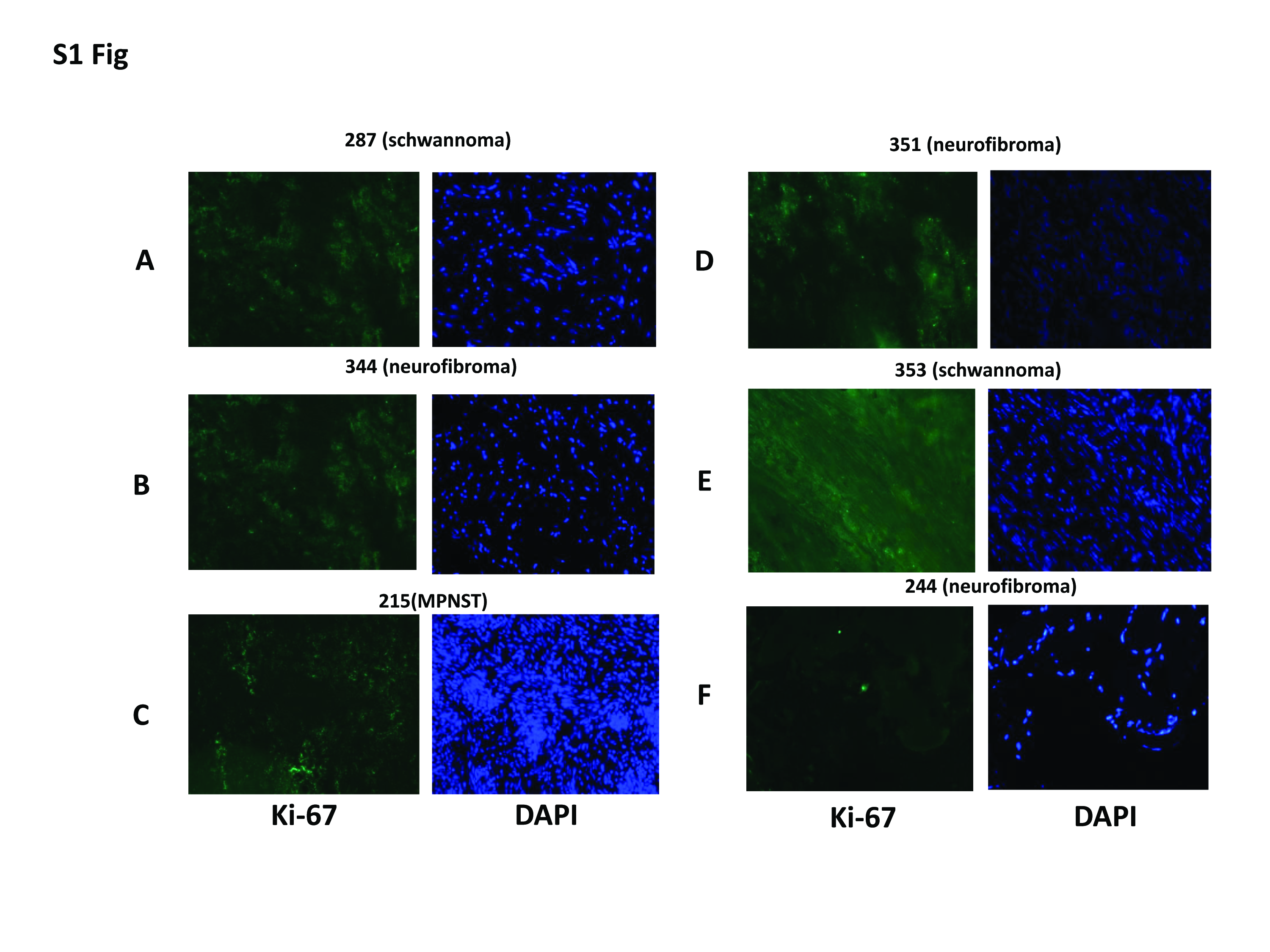

Supplement: S1 Fig — Ki-67 expression is evident in the neurofibromas, schwannomas and MPNSTs at variable levels (panel A-F). The Alexa Fluor 488 (green) staining represents the expression of Ki-67 and the DAPI (blue) stains the nucleus. (TIF) [file pone.0181529.s001.TIF]

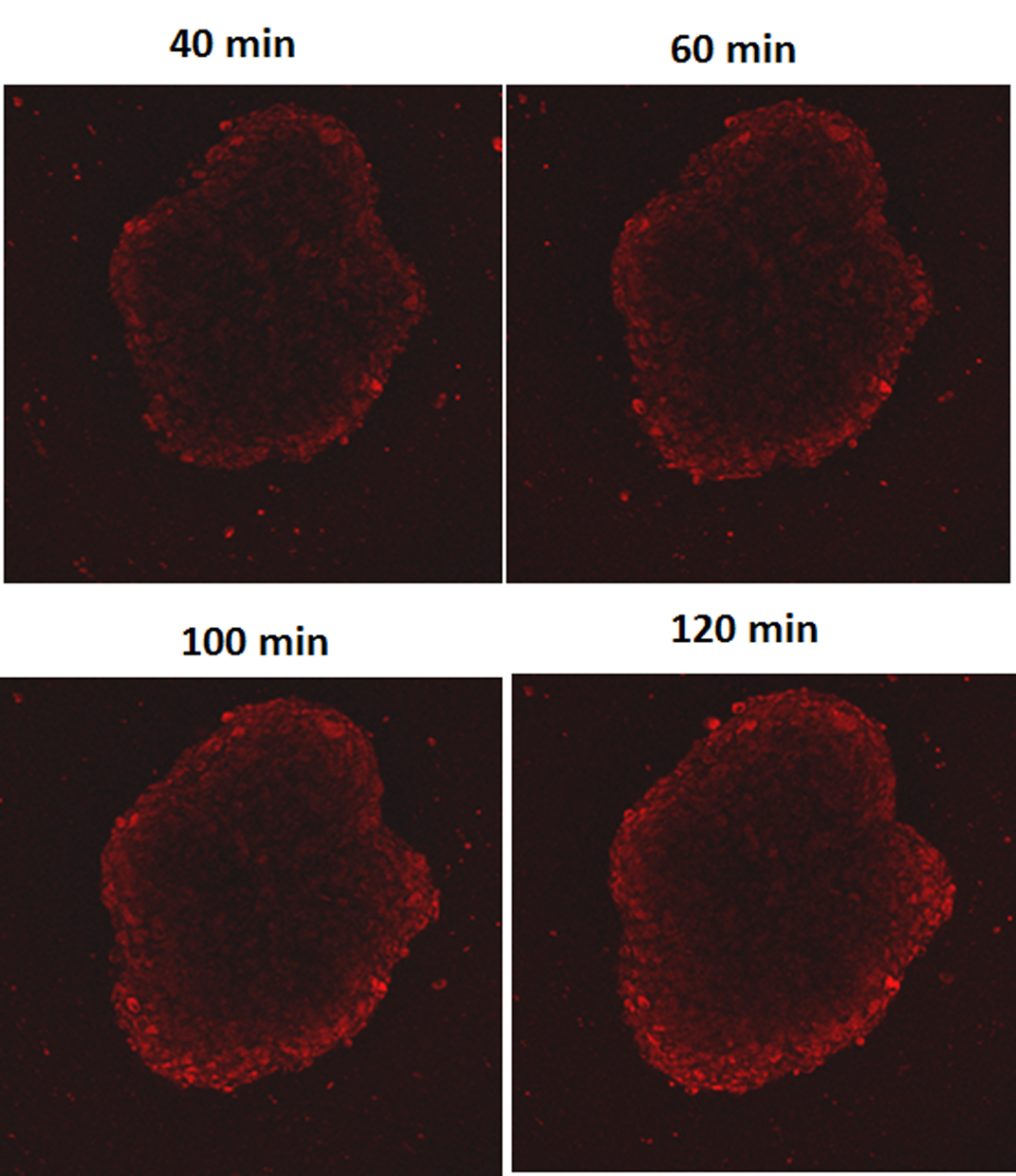

Supplement: S2 Fig — The figure indicates the projection maximum from the z-stack images at various time intervals. The endogenous fluorescence (red) of the IL13LIPDXR was utilized here to probe the intraspheroidal transport and diffusion into the cells. (TIF) [file pone.0181529.s002.tif]
