# Supplementary material for: Tumor targeted delivery of doxorubicin in malignant peripheral nerve sheath tumors
Source: PLoS One. 2018 Jan 5;13(1):e0181529. doi: 10.1371/journal.pone.0181529 (PMC5755733; doi:10.1371/journal.pone.0181529)
Supplement: S1 Raw Data — (DOCX) [file pone.0181529.s003.docx]

**S1 Raw Data**

**Table 1A.** **Raw data from serum chemistry analysis**

|  |  | **control** |  |  | **Non-targeted** | |  | **Targeted** |  |
| --- | --- | --- | --- | --- | --- | --- | --- | --- | --- |
| **BUN** | 25 | 20 | 21 | 65 | 24 | 29 | 24 | 19 | 23 |
| **Alk.Phos** | 52 | 63 | 67 | 55 | 67 | 67 | 53 | 77 | 67 |
| **AST** | 63 | 87 | 72 | 355 | 191 | 189 | 294 | 346 | 264 |
| **ALT** | 30 | 17 | 18 | 41 | 46 | 32 | 70 | 106 | 66 |

*Data analysis using Graphpad:*

| Parameter | Value |  |  |  |
| --- | --- | --- | --- | --- |
| Table Analyzed | |  |  |  |
| Data 1 |  |  |  |  |
| One-way analysis of variance | | |  |  |
| P value | 0.9873 |  |  |  |
| P value summary | ns |  |  |  |
| Are means signif. different? (P < 0.05) | No |  |  |  |
| Number of groups | 3 |  |  |  |
| F | 0.01284 |  |  |  |
| R squared | 0.00849 |  |  |  |
|  |  |  |  |  |
| ANOVA Table | SS | df | MS |  |
| Treatment (between columns) | 0.002593 | 2 | 0.001296 |  |
| Residual (within columns) | 0.3028 | 3 | 0.1009 |  |
| Total | 0.3054 | 5 |  |  |

**Table 1B. Raw data from serum chemistry analysis**

|  |  | **control** |  |  | **Non-targeted** | |  | **Targeted** |  |
| --- | --- | --- | --- | --- | --- | --- | --- | --- | --- |
| **T.Bil** | 0.3 | 0.6 | 0.5 | 0.4 | 0.8 | 0.7 | 0.7 | 0.7 | 0.5 |
| **Creatinine** | 0.2 | 0.2 | 0.2 | 0.1 | 0.1 | 0.2 | 0.1 | 0.1 | 0.1 |

*Data analysis using Graphpad:*

| Parameter | Value |  |  |
| --- | --- | --- | --- |
| Table Analyzed | |  |  |
| Data 1 |  |  |  |
| One-way analysis of variance | | |  |
| P value | 0.5479 |  |  |
| P value summary | ns |  |  |
| Are means signif. different? (P < 0.05) | No |  |  |
| Number of groups | 3 |  |  |
| F | 0.6437 |  |  |
| R squared | 0.1251 |  |  |
|  |  |  |  |
| ANOVA Table | SS | df | MS |
| Treatment (between columns) | 11270 | 2 | 5635 |
| Residual (within columns) | 78790 | 9 | 8754 |
| Total | 90060 | 11 |  |
|  |  |  |  |

**Table 1C. RAW DATA from IN VIVO THERAPEUTIC EFFICACY IN SCIATIC NERVE TUMOR MODEL**

|  |  | | **control** | |  | |  | |  | |
| --- | --- | --- | --- | --- | --- | --- | --- | --- | --- | --- |
| **Weeks** | #1 | | #2 | | #3 | | #4 | |  | |
| 0 | 16200 | | 128000 | | 6350 | | 27000 | |  | |
| 1 | 4660 | | 46900 | | ND | | 25800 | |  | |
| 2 | 13700 | | 7270 | | 3640 | | 214000 | |  | |
| 3 | 436000 | | 1.62E+07 | | 11600 | | 3320000 | |  | |
| 4 | 3.23E+07 | | 4.43E+07 | | 33000 | | 1.97E+07 | |  | |
| 5 | 1.46E+08 | | 6.43E+07 | | 34000 | | Dead/sacrificed* | |  | |
| 6 | 1.60E+08 | | 2.01E+08 | | 228000 | | Dead/sacrificed* | |  | |
|  |  | |  | |  | |  | |  | |
|  |  | | **LIP-DXR** | |  | |  | |  | |
| **Weeks** | #5 | | #6 | | #7 | | #8 | |  | |
| 0 | 31500 | | 11100 | | 10700 | | 27000 | |  | |
| 1 | 1890 | | 54500 | | 2400 | | 25800 | |  | |
| 2 | ND** | | 36300 | | 7910 | | 214000 | |  | |
| 3 | ND** | | 126000 | | 3350 | | 3320000 | |  | |
| 4 | 14700 | | 509000 | | 16600 | | 1.97E+07 | |  | |
| 5 | 18800 | | 4610000 | | 62900 | | Dead/sacrificed | |  | |
| 6 | 118000 | | 4.20E+07 | | 474000 | | Dead/sacrificed | |  | |
|  |  |  | | **IL13-LIP-DXR** | | | |  | |  |
| **Weeks** | #9 | #10 | | #11 | | #12 | | #13 | |  |
| 0 | 22300 | 4730 | | 2850 | | 354000 | | 17600 | |  |
| 1 | 4660 | 2700 | | 25200 | | 7410 | | 547 | |  |
| 2 | 7200 | 1260 | | 44600 | | 1930 | | 544 | |  |
| 3 | ND** | 12400 | | 38600 | | 1370 | | 1510 | |  |
| 4 | 14700 | 6380 | | 23600 | | 6930 | | 1520 | |  |
| 5 | 1690000 | 851 | | 136000 | | 361000 | | 13800 | |  |
| 6 | 8540000 | 17600 | | 618000 | | 3920000 | | 34500 | |  |

| *sacrificed: The mice which had large tumor volumes and which were unable to walk due to tumor burden were  sacrificed following the advice from the veterenarians in the comparative medicine department at Penn State College  of Medicine | | | | | | | | | | | | | | | | | | | | |
| --- | --- | --- | --- | --- | --- | --- | --- | --- | --- | --- | --- | --- | --- | --- | --- | --- | --- | --- | --- | --- |
| **ND: not-detectable by IVIS instrument, #1, #2…#13 represents the mouse identification number. |  |  |  |  |  |  |  |  |  |  |  |  |  |  |  |  |  |  |  |  |
| *Data analysis using Graphpad:*   \| Parameter \| Value \|  \|  \| \| --- \| --- \| --- \| --- \| \| Table Analyzed \| \|  \|  \| \| Data 1 \|  \|  \|  \| \| One-way analysis of variance \| \| \|  \| \| P value \| 0.0885 \|  \|  \| \| P value summary \| ns \|  \|  \| \| Are means signif. different? (P < 0.05) \| No \|  \|  \| \| Number of groups \| 3 \|  \|  \| \| F \| 2.783 \|  \|  \| \| R squared \| 0.2362 \|  \|  \| \|  \|  \|  \|  \| \| Bartlett's test for equal variances \| \| \| \| \| Bartlett's statistic (corrected) \| 49.68 \|  \|  \| \| P value \| P<0.0001 \|  \|  \| \| P value summary \| *** \|  \|  \| \| Do the variances differ signif. (P < 0.05) \| Yes \|  \|  \| \|  \|  \|  \|  \| \| ANOVA Table \| SS \| df \| MS \| \| Treatment (between columns) \| 4.11E+15 \| 2 \| 2.06E+15 \| \| Residual (within columns) \| 1.33E+16 \| 18 \| 7.38E+14 \| \| Total \| 1.74E+16 \| 20 \|  \| | | |  |  |  |  |  |  |  |  |  |  |  |  |  |  |  |  |  |  |
